# Supplementary material for: Regulation of stress granule formation in human oligodendrocytes
Source: Nat Commun. 2024 Feb 19;15:1524. doi: 10.1038/s41467-024-45746-6 (PMC10876533; doi:10.1038/s41467-024-45746-6)
Supplement: Supplementary file 1 — Supplementary information [file 41467_2024_45746_MOESM1_ESM.pdf]

Supplementary information

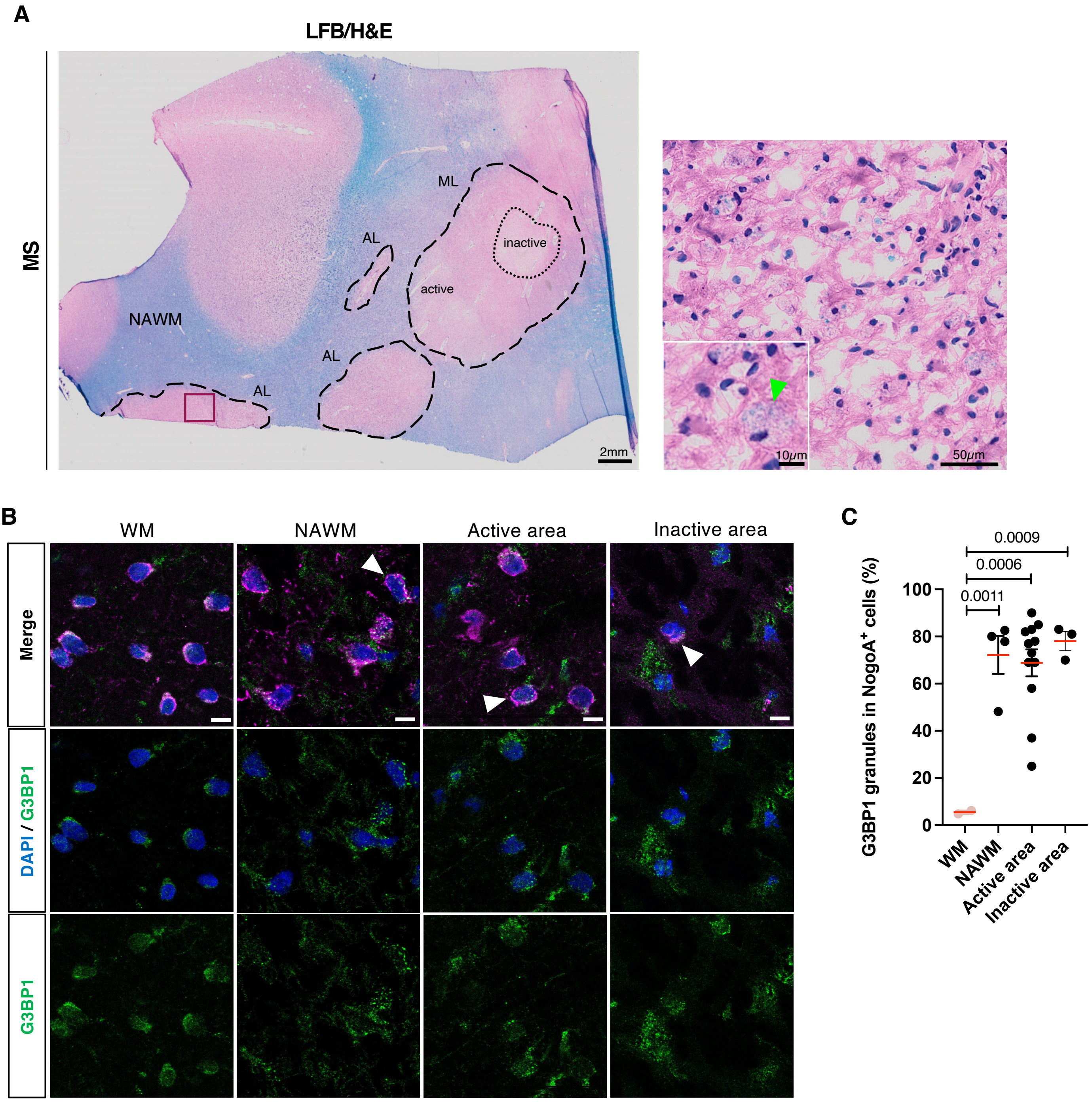

Figure S1: SGs expression in OLs in MS tissues.

(**A**) Tissue section of a RRMS case stained with LFB and H&E. Adjacent image outlines the active area of the mixed lesion showing the presence of LFB positive material in macrophages/microglia (green arrow). AL = active lesion; ML = mixed lesion.

(**B**) Representative confocal images of IHC staining for SGs (G3BP1^{+}) in OLs in control (WM) and MS (NAWM, active and inactive areas) tissue samples. G3BP1 granules are predominantly formed in the cytoplasm of OLs (arrowheads) of NAWM, active and inactive areas in MS patients. Sections were stained with DAPI (blue), NogoA (pink) and G3BP1 (green). Scale bars, 10µm.

(**C**) Quantification and gradient distribution of G3BP1^{+} oligodendrocytes were assessed in control and MS tissues. Data are graphed as the percentage of OLs positive for G3BP1 granules where the denominator is the number of OLs counted in that region (>100 cells when possible). Each dot in the graph represents a value from an individual and distinct area. Analyses done on 2 control patients accounting for independent regions as WM (n=2) and 3 MS patients accounting for NAWM (n=4), active (n=12) and inactive (n=3) areas.

All data are expressed as mean values ±SEM, analysed by one-way ANOVA followed by Bonferroni's multiple comparisons correction. All significant P-values are indicated; ns or unlabeled = not significant. WM = white matter; NAWM = normal appearing white matter; Active area = active areas of active & mixed MS lesion; Inactive area = inactive area of mixed MS lesion. Source data are provided as a Source Data file.

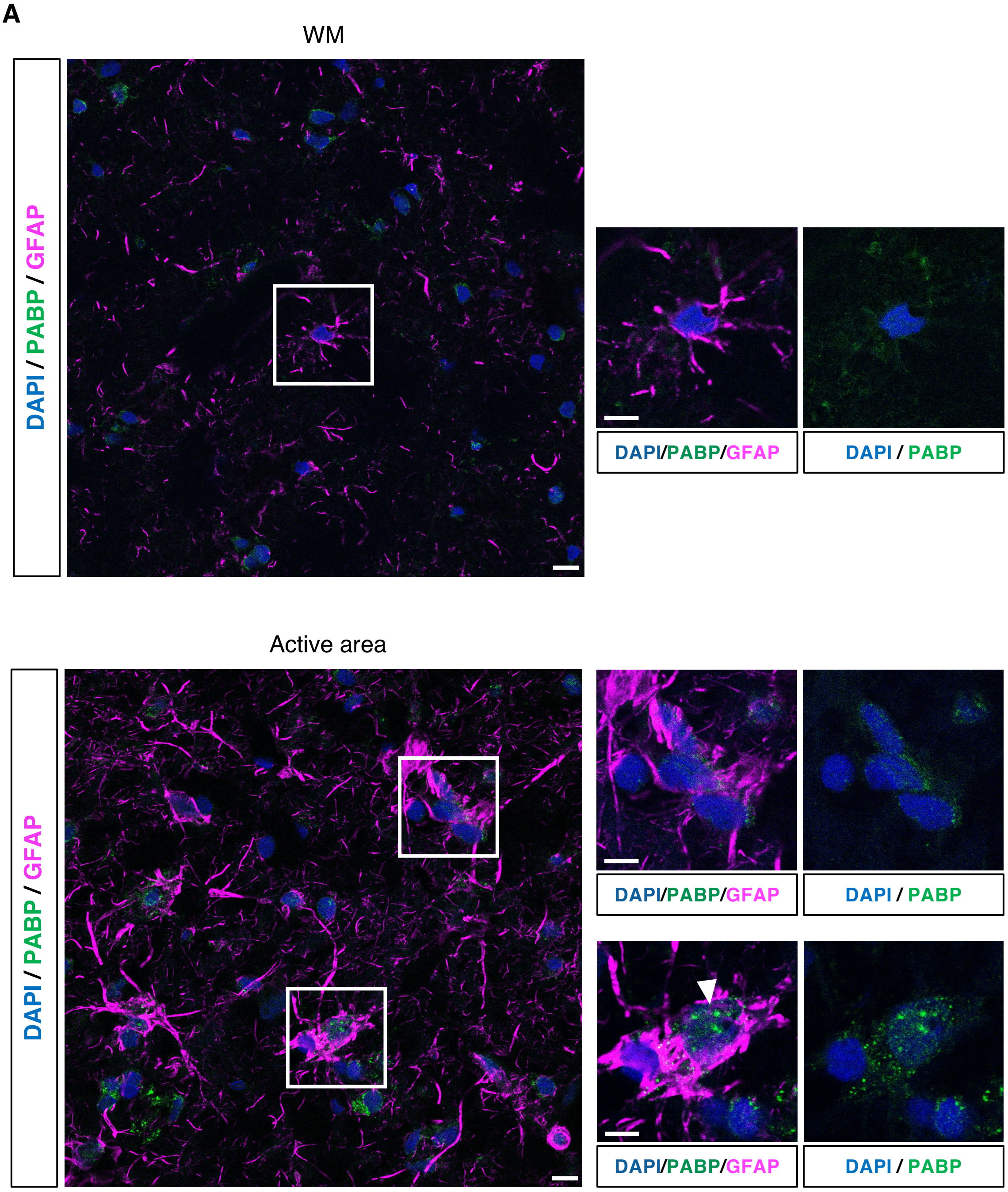

Figure S2: SGs expression in astrocytes in MS tissues.

(**A**) Representative confocal images of IHC staining for SGs (PABP^{+}) in astrocyte (GFAP^{+}) in control (WM) and MS (active area) patient tissues. PABP granules are formed in some astrocytes of active area of MS lesion. These SGs are present in the cytoplasm of the cell but also seems to form in the nucleus (arrowheads). Sections were stained with DAPI (blue), GFAP (pink) and PABP (green). For each staining, the SG experiments have been repeated 3 times in biologically independent samples, showing similar results. Scale bars, 20µm (low magnification); 10µm (high magnification).

All data are expressed as mean values ±SEM, analysed by one-way ANOVA followed by Bonferroni's multiple comparisons correction. All significant P-values are indicated; ns or unlabeled = not significant. WM = white matter; NAWM = normal appearing white matter; Active area = active areas of active & mixed MS lesion; Inactive area = inactive area of mixed MS lesion.

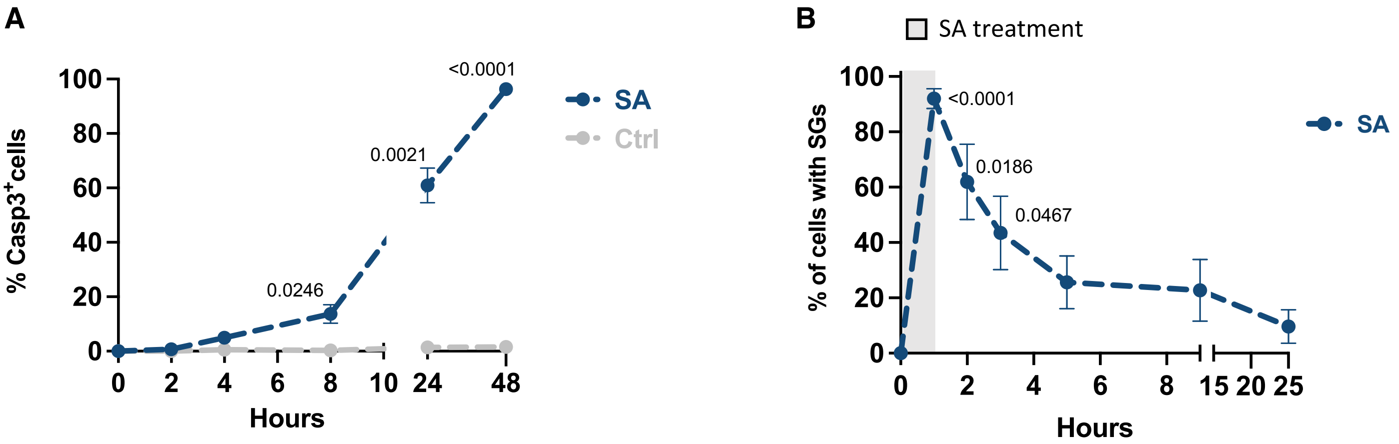

Figure S3: Recovery capacity of hOLs after an initial SA exposure.
(**A**) Quantitative analysis of apoptotic cell death (caspase 3^+) expressing hOLs over time under SA treatment (n=4). Apoptosis activation was determined by cleaved caspase 3/7 assay.
(**B**) Percentage of cells displaying SGs at different time points following 1 hour of SA exposure. Recovery was initiated by changing the SA treatment with optimal media (n=4).
Each dot in the graphs corresponds to an independent biological replicate. All data are expressed as mean values ±SEM, analyzed by paired two-tailed Student's *t*-test. All significant P-values are indicated; ns or unlabeled = not significant. Ctrl = optimal media; SA = sodium arsenite. Source data are provided as a Source Data file.

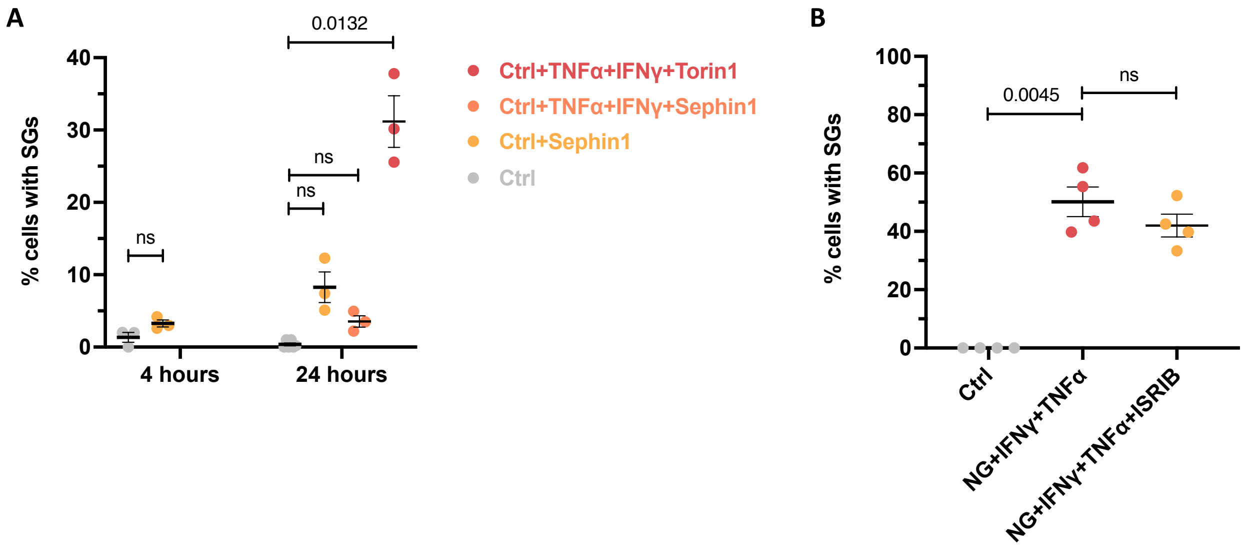

Figure S4: ISR and mTOR modulation and SG formation in human OLs.

(**A**) Quantitative analysis of human OLs exposed to Ctrl conditions, in the presence or absence of inflammatory cytokines and with addition of Sephin1 or Torin1 molecule for 4 and 24 hours. Each dot in the graphs corresponds to an independent biological replicate (n=3).

(**B**) Quantitative analysis of human OLs exposed to Ctrl or metabolic stress (NG) conditions, in the presence of inflammatory cytokines and with addition of ISRIB molecule for 24 hours. Each dot in the graphs corresponds to an independent biological replicate (n=4).

All data are expressed as mean values ±SEM, analyzed by ANOVA followed by Bonferroni's multiple comparisons correction. All significant P-values are indicated; ns or unlabeled = not significant. Ctrl = control (optimal) media; NG = no glucose. Source data are provided as a Source Data file.

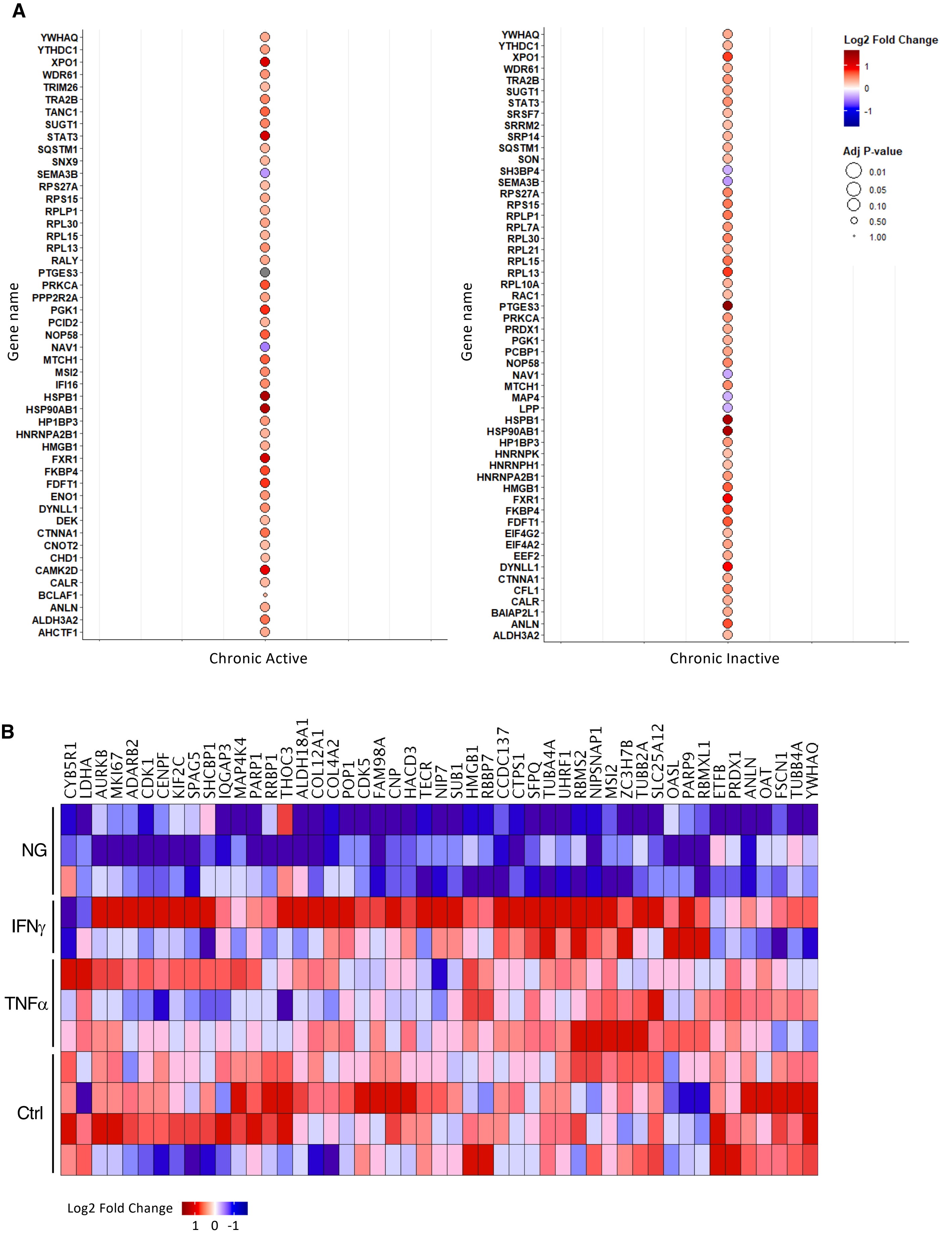

Figure S5: Transcriptional analysis of chronic active lesions and chronic inactive lesions in MS patients.
(**A**) Bubble plot of logFC depicting the SG-related genes in the chronic active and chronic inactive lesions, respectively. The blue-white-red scale indicates logFC expression as compared to Ctrl group; the size of the bubble denotes adjusted p-value.
(**B**) Bulk RNA sequencing analysis of the SG-related genes in hOLs after 2 days of treatment of TNFα (n=3), IFNγ (n=2) or NG (n=3) conditions using the list established by the Wang group. Heat maps shows the logFC of significant downregulated genes in NG conditions amongst the different treatments. Columns indicate individual samples grouped by treatment; blue-white-red scale indicates logFC expression as compared to corresponding control condition. Source data are provided as a Source Data file.

Supplementary Table 1: Clinical details of human brain tissue samples used for *in situ* investigations.

SAMPLE	AGE	SEX	DISEASE TYPE
**CTRL 1**	61	M	Tumour resection
**CTRL 2**	67	M	No neurological disease
**MS 1**	26	M	RRMS
**MS 2**	33	F	RRMS
**MS 3**	48	M	SPMS

F=Female; M=Male
SPMS=secondary progressive multiple sclerosis; RRMS=relapsing-remitting multiple sclerosis

Supplementary Table 2: Clinical details of human brain tissue samples used for *in vitro* functional investigations.

SAMPLE	AGE	SEX	DIAGNOSIS
1	1	F	Epilepsy
2	2	M	Focal cortical dysplasia IIA
3	4	M	Focal cortical dysplasia
4	6	M	Focal cortical dysplasia IIA
5	6	M	Epilepsy
6	7	M	Focal cortical dysplasia IIA
7	8	F	Focal cortical dysplasia
8	10	M	Focal cortical dysplasia IIA
9	10	M	Focal cortical dysplasia IIA
10	12	F	Focal cortical dysplasia IIA
11	12	M	Focal cortical dysplasia
12	12	M	Focal cortical dysplasia IIA
13	13	M	Focal cortical dysplasia
14	13	M	Focal cortical dysplasia IIA
15	13	F	Focal cortical dysplasia IIA
16	13	M	Epilepsy
17	14	M	Focal cortical dysplasia IIA
18	15	F	Epilepsy
19	18	M	Focal cortical dysplasia
20	18	F	Focal cortical dysplasia
21	21	F	Focal cortical dysplasia
22	26	M	Focal cortical dysplasia
23	37	F	Focal cortical dysplasia
24	37	F	Ganglioglioma
25	39	F	Post-focal cortical dysplasia
26	44	F	Mesial temporal sclerosis
27	51	F	Glioma
28	53	M	Gliosis
29	58	M	Glioma
30	60	M	Trigeminal Neuralgia
31	65	F	Focal cortical dysplasia
32	68	M	Glioma

Supplementary Table 3: Clinical details of human brain tissue samples used for molecular investigations.

SAMPLE	AGE	SEX	DIAGNOSIS	RNA SEQ STUDIES			
				CTRL	NG	IFNγ	TNFα
**1**	26	F	Gliosis	X	X		
**2**	31	F	Gliosis	X		X	X
**3**	41	M	Gliosis	X	X		X
**4**	43	M	Cortical dysplasia	X		X	
**5**	70	M	Cavernous angioma	X	X		X
