# Supplementary material for: Regulation of stress granule formation in human oligodendrocytes
Source: Nat Commun. 2024 Feb 19;15:1524. doi: 10.1038/s41467-024-45746-6 (PMC10876533; doi:10.1038/s41467-024-45746-6)
Supplement: Supplementary file 3 — Reporting Summary [file 41467_2024_45746_MOESM3_ESM.pdf]

nature portfolio

Corresponding author(s): Florian Pernin

Last updated by author(s): Jan 24, 2024

Reporting Summary

Nature Portfolio wishes to improve the reproducibility of the work that we publish. This form provides structure for consistency and transparency in reporting. For further information on Nature Portfolio policies, see our Editorial Policies and the Editorial Policy Checklist.

Statistics

For all statistical analyses, confirm that the following items are present in the figure legend, table legend, main text, or Methods section.

n/a	Confirmed	
☐	☒	The exact sample size (*n*) for each experimental group/condition, given as a discrete number and unit of measurement
☐	☒	A statement on whether measurements were taken from distinct samples or whether the same sample was measured repeatedly
☐	☒	The statistical test(s) used AND whether they are one- or two-sided<br>*Only common tests should be described solely by name; describe more complex techniques in the Methods section.*
☒	☐	A description of all covariates tested
☐	☒	A description of any assumptions or corrections, such as tests of normality and adjustment for multiple comparisons
☐	☒	A full description of the statistical parameters including central tendency (e.g. means) or other basic estimates (e.g. regression coefficient) AND variation (e.g. standard deviation) or associated estimates of uncertainty (e.g. confidence intervals)
☐	☒	For null hypothesis testing, the test statistic (e.g. *F*, *t*, *r*) with confidence intervals, effect sizes, degrees of freedom and *P* value noted<br>*Give P values as exact values whenever suitable.*
☒	☐	For Bayesian analysis, information on the choice of priors and Markov chain Monte Carlo settings
☒	☐	For hierarchical and complex designs, identification of the appropriate level for tests and full reporting of outcomes
☐	☒	Estimates of effect sizes (e.g. Cohen's *d*, Pearson's *r*), indicating how they were calculated

Our web collection on statistics for biologists contains articles on many of the points above.

Software and code

Policy information about availability of computer code

Data collection

Bulk RNAseq was performed at the Génome Québec Centre using the Illumina platform and NovaSeq 6000 PE100 machine

Data analysis

Bulk sequencing - We used the GenPipes workflow for alignment to the GRCh38 human genome and read counting. Raw FastQ files were aligned to the GRCh38 genome reference using the STAR aligner with default parameters. Raw reads were quantified using HTSeq count. Raw read counts were then normalized and variance-stabilized. DESeq2 package in R was employed for further analysis. Significantly differentially expressed genes were filtered using a p-value cutoff of <0.05. Heatmaps were generated after clustering using the Hierarchical Clustering Image function in GenePattern (v3.9.11). Gene clustering was performed using Pearson correlation and the normalized values were transformed using a long transformation. Finally, row normalization was applied for the final visualization.

Single nuclear RNA sequencing - The snRNAseq data of human MS and control patients were obtained from Schirmer, Jakel and Absinta datasets. The datasets were subjected to the standard Seurat pipeline for dimensionality reduction, cell type identification, gene expression normalization, and clustering. OL clusters were chosen in each dataset using canonical OL markers and information provided by the reference publications. Average expression and differentially expressed genes (DEG) between MS and control OLs were identified using a log2 (fold change) > 0.25 and an adjusted p-value < 0.05 as cutoffs. The log2FC and adjusted p-values were utilized to generate a bubble plot using ggplot2 v3.3.6 in R. A Venn diagram was employed to visualize the overlap between significant genes in the edge of chronic active and the edge of chronic inactive lesions using the Venny 2.1 web tool.

For manuscripts utilizing custom algorithms or software that are central to the research but not yet described in published literature, software must be made available to editors and reviewers. We strongly encourage code deposition in a community repository (e.g. GitHub). See the Nature Portfolio guidelines for submitting code & software for further information.

Data

Policy information about availability of data

All manuscripts must include a data availability statement. This statement should provide the following information, where applicable:

- Accession codes, unique identifiers, or web links for publicly available datasets
- A description of any restrictions on data availability
- For clinical datasets or third party data, please ensure that the statement adheres to our policy

All data generated or analyzed during this study are included in this article and its supplementary information files. Bulk sequencing data from this study have been deposited into the Gene Expression Omnibus (GEO) database (GSE249381).
Publicly available single nuclear RNA sequencing data are available under: NCBI Bioproject ID: 544731; Accession number GSE118257; Accession number GSE180759.

Research involving human participants, their data, or biological material

Policy information about studies with human participants or human data. See also policy information about sex, gender (identity/presentation), and sexual orientation and race, ethnicity and racism.

Reporting on sex and gender	Sex is mentioned in our report and gender is not available to us.
Reporting on race, ethnicity, or other socially relevant groupings	Surgical samples are determined by case selection from neurosurgical colleagues and not dependant on race or ethnicity.
Population characteristics	Age and sex of patients from which surgical samples were derived are provided in the Supplementary Tables.
Recruitment	Recruitment was based by surgical selection of human cases.
Ethics oversight	The use of human rapid post-mortem brain tissue samples was obtained with full ethical approval and informed consent from the Neuroimmunology Research Laboratory, Centre de Recherche du Centre Hospitalier de l'Université de Montréal (CRCHUM). The use of adult surgical samples was approved by the Montreal Neurological Institute Research Ethics Board and the use of pediatric surgical samples by the Montreal Children's Hospital Research Ethics Board.

Note that full information on the approval of the study protocol must also be provided in the manuscript.

Field-specific reporting

Please select the one below that is the best fit for your research. If you are not sure, read the appropriate sections before making your selection.

☒ Life sciences ☐ Behavioural & social sciences ☐ Ecological, evolutionary & environmental sciences

For a reference copy of the document with all sections, see nature.com/documents/nr-reporting-summary-flat.pdf

Life sciences study design

All studies must disclose on these points even when the disclosure is negative.

Sample size	Sample size reflected availability of material.
Data exclusions	No data were excluded.
Replication	Only single samples are available from surgical or autopsy materials.
Randomization	All suitable surgical samples were included in the study.
Blinding	Data analysis were derived by blinded observers for the entire study.

Reporting for specific materials, systems and methods

We require information from authors about some types of materials, experimental systems and methods used in many studies. Here, indicate whether each material, system or method listed is relevant to your study. If you are not sure if a list item applies to your research, read the appropriate section before selecting a response.

Materials & experimental systems

n/a	Involved in the study
☐	☒ Antibodies
☒	☐ Eukaryotic cell lines
☒	☐ Palaeontology and archaeology
☒	☐ Animals and other organisms
☒	☐ Clinical data
☒	☐ Dual use research of concern
☒	☐ Plants

Methods

n/a	Involved in the study
☒	☐ ChIP-seq
☒	☐ Flow cytometry
☒	☐ MRI-based neuroimaging

Antibodies

Antibodies used

Immunohistochemistry - Sudan Black solution was purchased from Millipore Sigma (#S2380). Anti-Nogo-A (11C7) antibody was provided by the Brain Research Institute, University of Zurich (17356385). G3BP1 (#181150), PABP (#21060), ATF4 (#31390) and anti-GFAP (#ab4674) antibodies were purchased from Abcam. p4E-BP1 (#2855S) antibody from Cell Signaling.

Immunocytochemistry - Anti-O4 antibody (#MAB1326) was purchased from RnDsystems. Anti-mouse (#56574) or anti-rabbit (#181150) G3BP1, PABP (#21060) and hnRNP A1 (#4791) antibodies were purchased from Abcam. Phosphorylated eIF2 (#701268) and phosphorylated 4E-BP1 (#2855S) from ThermoFisher and Cell Signaling, respectively. TDP-43 (#10782-2-AP) antibody was purchased from Proteintech.

Secondary antibodies - Goat anti-rabbit (#A11034) and anti-mouse (#A21121) Alexa Fluor 488; goat anti-rabbit (#A21428) Alexa Fluor 555 and Hoescht (#33258) were purchased from Invitrogen. Alexa Fluor 647 (#1021-31) from Southern Biotech and goat anti-rabbit CY3 (#111-166-047) from Jackson ImmunoResearch.

Validation

Validation of commercial antibodies used in this study were done by the manufacturer and details of these validation efforts are described on the manufacturer's website as detailed below.

Immunohistochemistry:
anti-G3BP1 (#181150, Abcam)
https://www.abcam.com/products/primary-antibodies/g3bp-antibody-epr13986b-ab181150.html
anti-PABP (#21060, Abcam)
https://www.abcam.com/products/primary-antibodies/pabp-antibody-ab21060.html
anti-ATF4 (#31390, Abcam)
https://www.abcam.com/products/primary-antibodies/atf-4-antibody-ab31390.html
anti-GFAP (#ab4674, Abcam)
https://www.abcam.com/products/primary-antibodies/gfap-antibody-ab4674.html
anti-p4E-BP1 (#2855S, Cell signaling technology)
https://www.cellsignal.com/products/primary-antibodies/phospho-4e-bp1-thr37-46-236b4-rabbit-mab/2855

IImmunocytochemistry:
anti-O4 antibody (#MAB1326, RnDsystems)
https://www.rndsystems.com/products/human-mouse-rat-chicken-oligodendrocyte-marker-o4-antibody-o4_mab1326
anti-G3BP1 (#56574, Abcam)
https://www.abcam.com/products/primary-antibodies/g3bp-antibody-2f3-ab56574.html
anti-hnRNP A1 (#4791, Abcam)
https://www.abcam.com/products/primary-antibodies/hnrnp-a1-antibody-ab4791.html
anti-phosphorylated eIF2alpha (#701268, Thermofisher)
https://www.thermofisher.com/antibody/product/Phospho-EIF2S1-Ser51-Antibody-clone-10H21L20-Recombinant-Monoclonal/701268
anti-phosphorylated 4E-BP1 (#2855S, ThermoFisher)
https://www.thermofisher.com/antibody/product/Phospho-4EBP1-Thr37-Thr46-Antibody-Polyclonal/PA5-77963
anti-TDP-43 (#10782-2-AP, Proteintech)
https://www.ptglab.com/products/TARDBP-Antibody-10782-2-AP.htm

Secondary antibodies
Goat anti-rabbit Alexa Fluor 488(#A11034, Thermofisher)
https://www.thermofisher.com/antibody/product/Goat-anti-Rabbit-IgG-H-L-Highly-Cross-Adsorbed-Secondary-Antibody-Polyclonal/A-11034
Goat-anti-mouse Alexa Fluor 488(#A21121, Thermofisher)
https://www.thermofisher.com/antibody/product/Goat-anti-Mouse-IgG1-Cross-Adsorbed-Secondary-Antibody-Polyclonal/A-21121
Goat anti-rabbit Alexa Fluor 555 (#A21428, Thermofisher)
Hoescht (#33258, Thermofisher)
https://www.thermofisher.com/order/catalog/product/H3569

Goat anti-rabbit Alexa Fluor 647 (#1021-31, Southern Biotechnology)
https://www.southernbiotech.com/goat-anti-mouse-igm-af647-1021-31
Goat anti-rabbit CY3 (#111-166-047, ackson ImmunoResearch)
https://www.jacksonimmuno.com/catalog/products/111-166-047
